# Elimination of *gambiense* human African trypanosomiasis as a public health problem in Republic of Guinea: Paving the way for zero transmission

**DOI:** 10.1371/journal.pntd.0014469

**Published:** 2026-07-01

**Authors:** Moïse Kagbadouno, Oumou Camara, Bailo Mamadou Diallo, Abdoulaye Dansy Camara, Aissata Soumah, Mamadou Leno, Favié Béavogui, Mohamed Diaby Gassama, Issiaga Camara, Bamoro Coulibaly, Dramane Kaba, Aïssata Camara, Salimatou Boiro, Hamidou Ilboudo, Jean-Baptiste Rayaisse, Annette MacLeod, Joseph Ndungu, Sylvain Bieler, Paul Bessell, Damien Ott, Veerle Lejon, Sophie Ravel, Fabrice Courtin, Philippe Solano, Vincent Jamonneau, Jean-Mathieu Bart, Brice Rotureau, Bruno Bucheton, Mamadou Camara

**Affiliations:** 1 Programme National de Lutte contre les Maladies Tropicales Négligées, Ministère de la Santé, Conakry, Guinea; 2 University Gamal Abdel Nasser of Conakry, Conakry, Guinea; 3 Institut Pierre Richet (IPR), Bouaké, Côte d’Ivoire; 4 Parasitology Unit, Institut Pasteur of Guinea, Conakry, Guinea; 5 Institut de Recherche en Sciences de la Santé (IRSS)/Unité de Recherche Clinique de Nanoro, Nanoro, Burkina Faso; 6 CIRDES, Bobo-Dioulasso, Burkina-Faso; 7 University of Glasgow, Scotland, United Kingdom; 8 Foundation Initiative for New Diagnostics (FIND), Geneva, Switzerland; 9 Independent Consultant, Edinburgh, United Kingdom; 10 Ambassade de France, Conakry, Guinea; 11 INTERTRYP, Université de Montpellier, CIRAD, IRD, Montpellier, France; University of Antwerp Drie Eiken Campus: Universiteit Antwerpen Campus Drie Eiken, BELGIUM

## Abstract

The Republic of Guinea has faced an important challenge with human African trypanosomiasis (HAT), which was endemic over the last century. After initial control in the 1960s–1970s, HAT resurged in the 1990s along the Guinean coast, driven by economic and demographic pressures on the mangrove ecosystem. In response, the Guinean government established a national control program in 2002, focusing on medical mass screenings. In 2012, vector control using tiny targets was introduced in the East Boffa focus to reduce fly density and human-vector contact. However, the Ebola epidemic from 2013 to 2016 disrupted these efforts, leading to a reliance on passive screening. Resuming screenings in 2016–2017 revealed increased cases in all foci except the East Boffa area, where vector control had been effective. Vector control continued during the SARS-CoV2 pandemic and at the same time targeted door-to-door screenings were introduced to target high-risk individuals. Since 2018, around 30,000 at-risk individuals have been screened annually. These strategies reduced the total number of new cases below 1 per 10,000 inhabitants in endemic areas over the period 2019–2023, allowing to validate the elimination of HAT as a public health problem. The Guinean team and partners then focused on systematic spatial monitoring of patients and community engagement in vector control. The program also integrates control of other neglected tropical diseases and addresses new research questions, especially about anatomical and animal reservoirs for parasites. These efforts, combined with implementation of improved diagnostic tests and new oral treatments, through active involvement in multiple clinical trials and studies, now aim to interrupt HAT transmission by 2030.

## Introduction

On January 18th, 2025, the World Health Organization (WHO) validated the elimination of human African trypanosomiasis (HAT) as a public health problem in Guinea. In this mini-review, we summarized some key facts that contributed to reach elimination following the WHO roadmap.

## The Republic of Guinea and its health system capabilities

**Fig 1:**
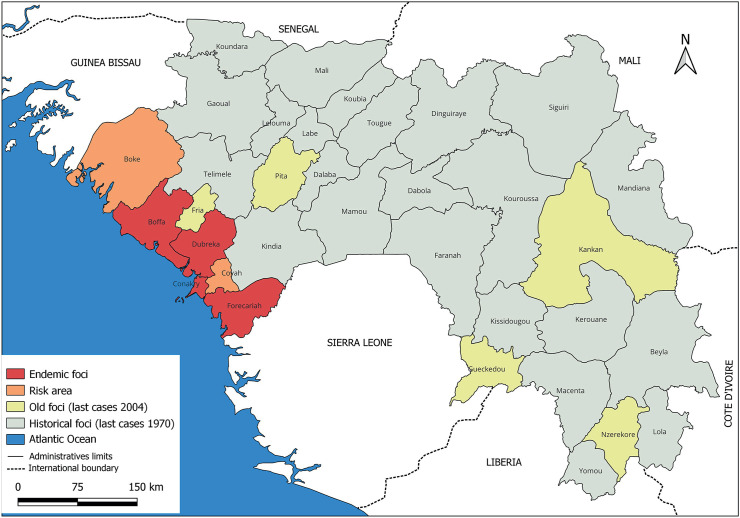
Administrative map of the Republic of Guinea showing endemic areas, old foci, historical foci and areas at risk of transmission (adapted from [1]). Geographical sub-divisions show Guinean prefectures.

The Republic of Guinea, a West African nation of 245,857 km^2^, is bordered by the Atlantic Ocean and six countries (Fig 1). It is divided into four natural regions and 33 prefectures [1]. With a population of nearly 14 million in 2024, the country is characterized by a density of 59 inhabitants per km^2^ and a predominantly young and rural population, though a large concentration (2.2 million) lives in the capital, Conakry. Guinea is rich in natural resources, including bauxite, iron, gold, and diamonds.

Despite recent increases in healthcare spending, Guinea’s health system remains fragile. It is structured into central, regional, and local levels, emphasizing decentralization and community participation across public, private, and community sectors. The country has 1,383 public health structures, over half of which do not meet WHO standards, and only 3,383 hospital beds for 14 million people (more than 4,000 per hospital bed). The health workforce of 8,005 personnel has decreased since 2014 due to retirements.

The primary health challenges are communicable diseases like malaria, HIV/AIDS, and tuberculosis, alongside a growing concern for non-communicable diseases. Past epidemics, notably Ebola and Covid-19, have exposed the system’s vulnerabilities [2]. While Guinea has made substantial progress in health crisis management, major challenges persist in infrastructure, human resources, and health coverage [3].

## Historical data and endemic areas

HAT has been a long-standing endemic disease in West Africa [4]. The disease emerged as an epidemic in the late 19th and early 20th centuries, affecting Senegal, Mali, Niger, Burkina Faso and Guinea by 1920 [5,6]. In Guinea, HAT was recognized as early as 1902, with Upper Guinea noted as one of the most impacted regions [7]. Gustave Martin documented numerous villages affected by the disease in 1906 [8]. Despite early recognition, surveillance and control activities were poorly structured until the 1930s [6]. From 1939 onwards, surveillance intensified, and autonomous programs were established to screen and treat HAT patients. High numbers of infections were reported in regions like Kissidougou and Guéckédou during the 1939–1940 period, with over 10,000 cases in Guinea by 1941 [6,9,10]. Control efforts in the 1940s led to a reduction in prevalence, with only 865 HAT cases reported in 1961 [11]. However, the disease persisted, particularly in the savanna zone and specific foci like Kissidougou [6,9,12]. From the 1970s, HAT cases resurged in Guinea, not in the historical foci, but in a new ecological context in the mangrove of maritime Guinea [6] and by the late 1990s, Guinea became the most affected country in West Africa [9].

The decline and eventual disappearance of gHAT from historical foci in the Guinea’s forested regions occurred without clearly documented large-scale vector control measures, suggesting that human-centered medical interventions and ecological shifts played a decisive role [5,6]. The disappearance of gHAT from historical foci by the 1960s suggests that human-targeted strategies alone (mass screening, treatment, and case isolation) were sufficient to interrupt transmission cycles in the absence of vector control. Ecological changes such as deforestation, agricultural expansion, and reduced human-tsetse contact (altered riverine habitats) may have also accidentally contributed to suppress vector populations. Human behavior shifts such as avoidance of tsetse-infested areas, reduced mobility in endemic zones, and/or unrecorded local vector suppression (e.g., bush clearing) could have also contributed. Underreporting and surveillance gaps may, however, not be excluded: some residual transmission may have persisted but gone undetected due to relaxed control efforts (1962–1971). The coastal resurgence in the 1970s–1990s and the occurrence of few sporadic non-coastal cases in historical foci (2003, 2004 and 2006) highlight that gHAT elimination is fragile without sustained surveillance, especially in areas where vector control was never systematically applied [6].

Since 2006, the endemic area of in Guinea consists of three main foci: Boffa, Dubréka, and Forécariah (Fig 1). These areas are characterized by mangrove landscapes where exposure to tsetse flies is high [13]. The epidemiology of HAT in Guinea is linked to anthropogenic pressure, with activities such as agriculture, fishing, wood cutting and salt extraction increasing exposure to the tsetse and thus *Trypanosoma brucei gambiense* (*T. b. gambiense*) infection [14]. Active screening campaigns since 2003 [2] and the introduction of vector control in 2012, first in the East Boffa area [15], markedly reduced the HAT prevalence. However, the 2013–16 Ebola epidemic resulted in increased transmission, making Guinea the second-most affected country in Africa after the Democratic Republic of the Congo (DRC) in terms of HAT cases [16]. Post-Ebola strategies, combining active and passive case detection and vector control in all endemic areas, then resulted in an over 70% decrease in prevalence [1].

Three cases of HAT imported from Guinea were reported in Europe in 2017, 2018, and 2020, involving individuals who frequently visited endemic zones [17]. These cases highlight the potential international dimension of the disease and the need for continued vigilance.

## Control and surveillance activities: An evolving partnership for HAT elimination

In 2002, the Guinean government, with support from the WHO and experts, drafted a policy to address HAT, which had become a significant public health issue. The Programme National de Lutte contre la Trypanosomiase Humaine Africaine (PNLTHA) was established to coordinate control efforts, focusing on the endemic areas, namely Dubréka, Forécariah, and Boffa (Fig 1). The PNLTHA evolved through various phases, integrating new strategies and actors, such as entomologists and community agents. Its aim was to sustainably eliminate HAT. In 2022, the PNLTHA was integrated into the Programme National de Lutte contre les Maladies Tropicales Négligées à Prise en Charge des Cas (PNLMTN-PCC), focusing on case management of different neglected tropical diseases (NTD). The HAT Unit of the PNLMTN-PCC comprises personnel from various ministries, including the Ministry of Health and Public Hygiene, the Ministry of Higher education, Research and Technology and the Ministry of Agriculture and Livestock. The team includes doctors, biologists, laboratory technicians, nurses, and support staff deployed across key locations. The PNLMTN-PCC, helped by research organizations, also trains students including its own human resources, enhancing its research and control capabilities. The program has developed strong partnerships with African and European research institutions, such as Institut Pierre Richet (IPR) in Cote d’Ivoire, Centre International de Recherche-Développement pour l’Elevage en zone Subhumide (CIRDES) in Burkina Faso, Institut de Recherche pour le Développement (IRD), Institut Pasteur, Institut Pasteur of Guinea, as well as University of Glasgow and University of Warwick. Partners also included international organizations like WHO, and public–private partnerships such as the Foundation for Innovative New Diagnostics (FIND), and the Drugs for Neglected Diseases Initiatives (DNDi). These collaborations have facilitated research on HAT control strategies, including patient screening, treatment, and vector control.

The program’s research aligns with national policies and with the WHO guidelines and roadmap on NTDs, aiming to eliminate HAT by 2030 [18]. The program combines medical and entomological control activities within a single framework, optimizing information sharing and coordination. Medical control involves screening and treating patients, while vector control focuses on deploying insecticide-impregnated tiny targets to reduce tsetse densities in areas of transmission [19]. These activities mostly supported by the Gates Foundation [20] and EU-EDCTP2 [21] since 2012, were planned during biannual steering committee meetings and documented in semi-annual and annual reports.

In the 2000s, passive surveillance was operational in former foci, and the algorithm used was initially relying on CATT. Since 2013, a new passive surveillance system has been implemented with RDT performed on clinically suspicious individuals. In the event of a positive RDT, blood is sampled on filter paper for trypanolysis test. This strategy has been implemented in 9 health centers that are supervised once a year, including capacity building of agents to optimize the effectiveness of this surveillance. In 2018, a case of positive trypanolysis was reported in a pregnant patient in Kankan. Targeted reactive screening (door-to-door) was organized around the case, and no other cases were diagnosed [22].

**Fig 2:**
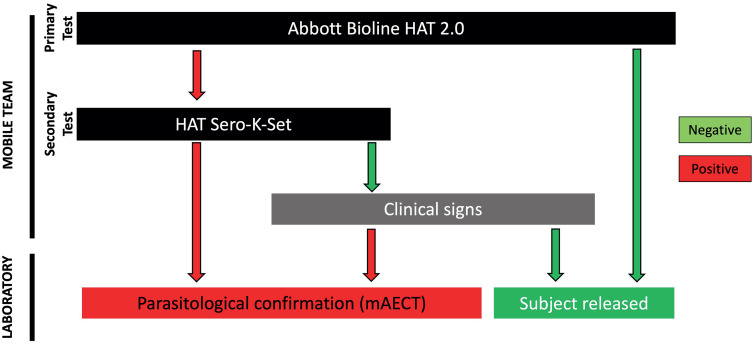
Current algorithm used by the PNLMTN-PCC to detect HAT cases by active screening in Guinea (adapted from [1]). mAECT: mini-anion exchange centrifugation test.

**Fig 3:**
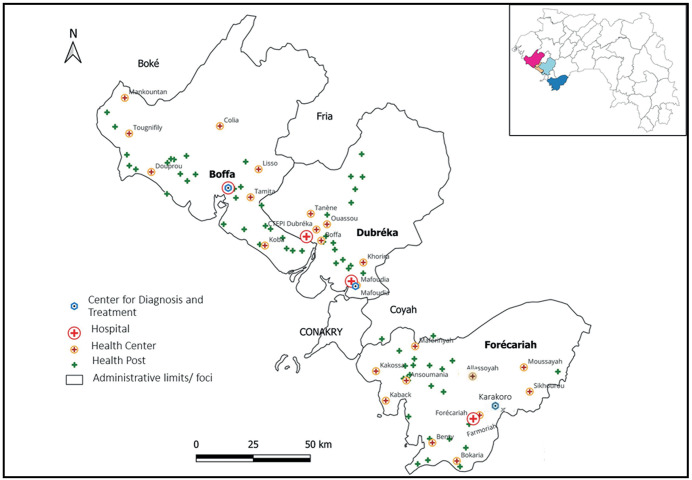
Network of health structures that are part of the passive screening strategy in the three endemic foci in Guinea. (Map: OSM; Projection: WGS 84 EPSG 4326; Data source: PNLTHA/IRD).

Active screening strategies were adapted to health crises like Ebola and COVID-19, as well as changes in disease prevalence by shifting from mass screenings using card agglutination tests for trypanosomiasis (CATT), to targeted door-to-door campaigns, using rapid diagnostic tests (RDTs) (Fig 2) [1]. Passive screening involves a network of 101 health centers where patients presenting with symptoms suggestive of HAT are tested using RDTs and referred for confirmation and treatment at three specialist laboratories (Fig 3) [2].

Confirmation in both active and passive surveillance is through screening vascular fluids for visible parasites: if swollen cervical lymph nodes are palpable, then aspirates are examined by microscopy, if not or if negative, then venous blood is treated using the mini-anion exchange centrifugation test (mAECT) and examined. Key therapeutic clinical trials demonstrating fexinidazole efficacy were partially carried out in Guinea [23]. From 2019 to 2023, 30.1% (45/147 HAT cases) of HAT patients were treated with fexinidazole, while advanced meningo-encephalitic stage patients still received nifurtimox-eflornithine combination therapy (NECT) (102/147 HAT cases). Treated patients were followed up for 24 months to monitor for relapses.

After diagnosis of serological suspects or confirmed cases, the PNLMTN-PCC deployed an innovative and adaptable targeted response, combining medical and vector control measures. Spatial monitoring of diagnosed HAT patients helped to identify at-risk locations for transmission [24], where insecticide-impregnated tiny targets are deployed, and reactive medical screenings were conducted [1]. This approach has proven effective in interrupting transmission and protecting exposed individuals [1].

## Epidemiology

HAT in Guinea has seen significant changes over the past two decades. From 2002 to 2023, a total of 1,703 new gambiense HAT (gHAT) cases were detected in Boffa (754 cases), Dubréka (550 cases), Forécariah (328 cases), but also in Conakry (43), Boké, Coyah, and Fria (12), as well as in Forest Guinea (16 cases, with the last detection in 2004). All these cases were parasitologically confirmed and immediately treated according to the WHO recommendations, with a minimal treatment refusal (*n* = 4) (PNLTHA and WHO official records) (https://apps.who.int/neglected_diseases/ntddata/hat/hat.html).

**Fig 4:**
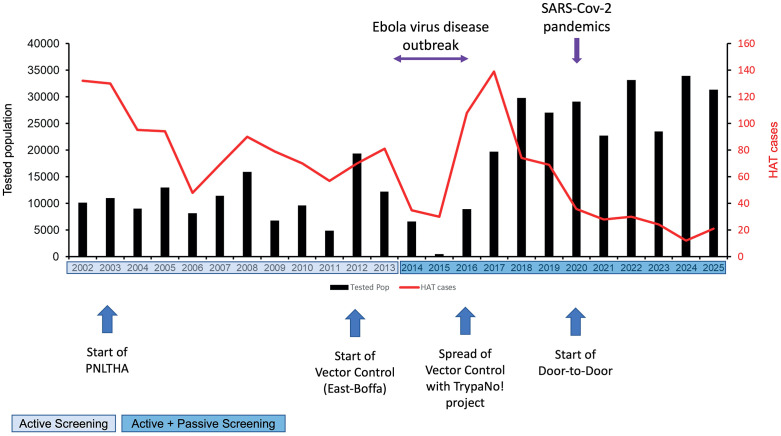
Evolution of the number of HAT cases against the screening strategies from 2002 to 2025.

**Fig 5:**
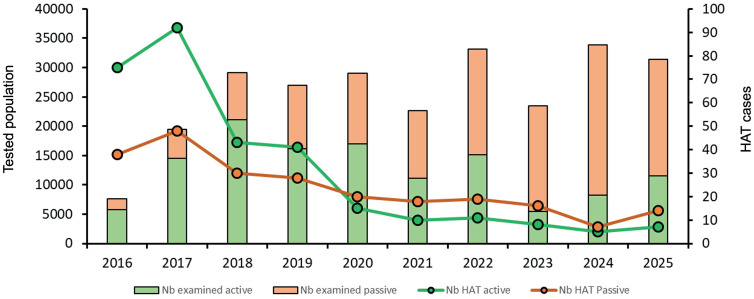
Evolution of the number of HAT cases by passive and active detection from 2016 to 2025.

Active screening was initially the main medical strategy, especially post-2002 with the establishment of PNLTHA. Then, passive screening was extended post-Ebola, with a network of peripheral health centers. Between 2002 and 2023, over 244,956 and 88,399 individuals were tested in active and passive screening, respectively, and half of the cases were detected by passive screening [2,21] (Figs 4 and 5). A high number of HAT cases was observed between 2002 and 2010, with 84 cases/year on average. Then, introduction of vector control (VC) in the 2010s led to a reduction in the number of cases, except during the Ebola epidemic (2013–2015). A sustained decrease was observed post-2018, with an average of 43 cases/year until 2023 (Figs 4 and 5). Interestingly, during this period, stage 2 cases were predominant (stage 2/stage 1 ratio = 4.6). In 2024 and 2025, only 12 and 21 cases were detected, respectively, and mostly in the Dubreka focus. The fact that no new stage 1 case was identified since 2021 is an intriguing observation. One hypothesis could be a reduced sensitivity for early-stage disease in the current diagnostic pathway. This is unlikely as the current routine diagnosis algorithm was optimized to detect all cases [21,1], and in case of doubts, a trypanolysis test is performed, and serosuspects are followed up until resolution [25]. The absence of new stage 1 cases would rather be an argument showing that the transmission intensity has significantly decreased and that the interruption of transmission is ongoing, with only residual stage 2 cases resulting from long-lasting infections being sporadically detected, in a context of long-term vector control.

**Fig 6:**
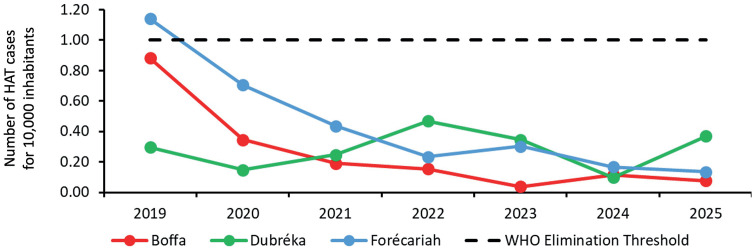
Number of cases per 10,000 inhabitants per focus from 2019 to 2025.

Over the 5 years between 2019 and 2023, a decrease from 69 HAT cases in 2019–23 cases in 2023 was reported under similar screening efforts (~27,000 individuals tested annually). In the meantime, the ratio of HAT cases detected through passive screening increased from 40% in 2019 to 65% in 2023. Following the vector control implementation in 2012 in Boffa and improved public awareness, a sharp decrease was seen with only a single case reported in 2023 (likely a rare case of mother-to-child transmission). In Dubréka and Forécariah, the decrease in prevalence was less pronounced, as vector control was implemented in 2016 and in 2018 respectively. The elimination threshold (less than 1 case per 10,000 inhabitants in endemic health districts) was reached in 2020 (Fig 6).

Despite these remarkable results, Guinea is still reporting the highest number of gHAT cases in West Africa. However, there is little surveillance in neighboring countries such as Sierra Leone, Liberia, and Guinea-Bissau, while Mali and Côte d’Ivoire report few or no cases. In 2023, passive surveillance has been established in Sierra Leone’s Kambia district, which is at the border of the Guinean Forécariah focus, with no confirmed cases so far.

## Vector control

Medical screenings alone are insufficient to halt transmission due to low coverage and population mobility [26]. Currently, in Guinea, the interaction between humans and the tsetse vector, *Glossina palpalis gambiensis*, primarily occurs in mangrove ecosystems where economic activities such as fishing and wood cutting are prevalent [13].

Since 2012, vector control (VC) reduced tsetse densities using deltamethrin-impregnated tiny targets [19]. This approach aims to decrease human-vector contact in high-risk transmission areas [27]. The proof of principle was demonstrated on the Loos Islands, where a combination of insecticide treatments and traps significantly reduced tsetse populations [28].

**Fig 7:**
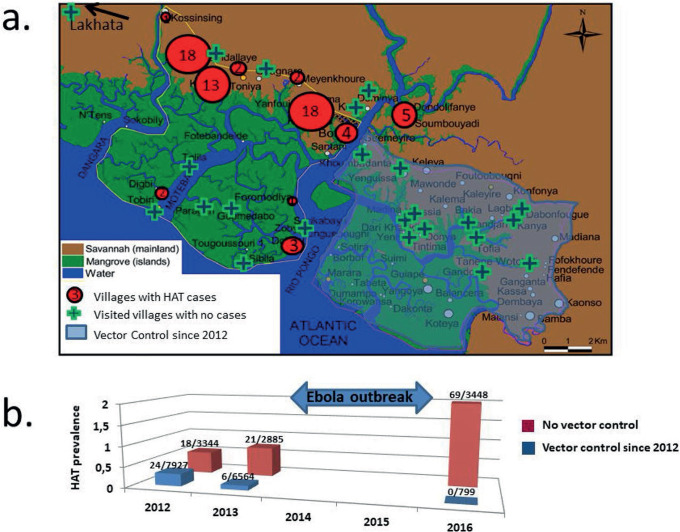
Impact of vector control in the Boffa focus (adapted from [16]). **a.** Geographic distribution of HAT cases diagnosed during two medical surveys led in the Boffa focus in 2016. **b.** Evolution of sleeping sickness prevalence in the Boffa focus assessed during active screening campaigns conducted in 2012, 2013 and 2016. The number of diagnosed HAT cases/number of persons screened is indicated above the histograms.

**Fig 8:**
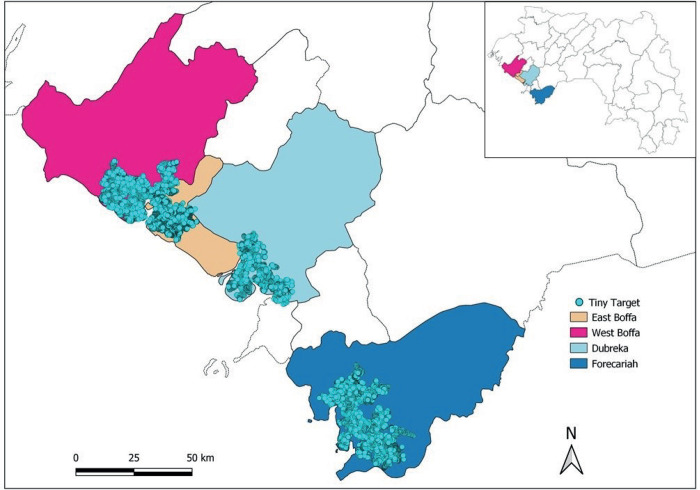
Tiny target deployment in coastal Guinea. Since 2012, thousands of tiny targets (blue circles) are deployed annually in the HAT transmission foci of West Boffa (pink), East Boffa (orange), Dubreka (light blue) and Forecariah (dark blue). (Map: OSM; Projection: WGS 84 EPSG 4326; Data source: PNLTHA/IRD).

Next, in a pilot phase (2012–2015), the deployment of tiny targets in the East Boffa area led to a 80% reduction in tsetse density and a significant decrease in HAT cases (from 0.3% to 0.1%; *p* = 0.01) [13]. The Ebola epidemic in 2014–2015 disrupted active screening in East Boffa, but community-led maintenance of tiny targets continued, showing sustained HAT control (Fig 7) [16]. After this success, a generalization phase (2016–2018) extended VC coverage to all active coastal foci, with up to 16,000 tiny targets deployed annually (Fig 8).

This led to marked reductions in tsetse densities as well as HAT cases across all intervention areas (PNLTHA and WHO official records). To maintain VC and optimize its cost, a maintenance phase (2019–2021) was then launched with the introduction of digital tools like Open Data Kit (ODK) and KoBoCollect to improve data collection and monitoring efficiency [29]. A progressive reduction phase (2022–2023) was recently applied to scale-down VC to a sustainable process: a targeted approach was introduced, focusing on community-led monitoring, as well as the use of ‘companion’ tiny targets in isolated areas. Despite a reduction in the number of tiny targets, control of tsetse densities was maintained.

Community involvement has been integral to the success of the VC strategy. Local agents have been trained to deploy and monitor screens, and community mobilization efforts have improved awareness and participation. This has led to sustainable control activities even during periods of disruption, such as the Ebola epidemic [16]. In total, the use of insecticide-impregnated tiny targets has resulted in substantial reductions in tsetse densities across all intervention areas. This has directly contributed to a decrease in HAT cases, demonstrating the effectiveness of the VC strategy (Camara AD *et al*, PLoS NTD, under review).

## Animal African trypanosomosis (AAT)

Livestock is an important Guinea’s rural sector, contributing importantly to poverty reduction and food security. It represents 4.9% of Guinea’s Growth Domestic Product (GDP), with over 80% of rural households owning livestock [30]. The primary species include cattle, sheep, goats, pigs, poultry, and equines. Notably, N’dama cattle and Djallonké sheep and goats dominate due to their hardiness and trypanotolerance. Regions like Kankan, Boké, and Labé lead in livestock farming. Pig farming is concentrated in Nzérékoré and Conakry. Guinea faces challenges with transboundary animal diseases and zoonoses, which hinder livestock development. Veterinary services and epidemiological surveillance are weak, with limited access to veterinary inputs and information. AAT is not considered as a major veterinary problem in Guinea, leading to limited data and investment. The g-HAT active foci of coastal Guinea do not harbor high densities of livestock, including cattle. Studies in Lower, Upper, and Forest Guinea regions have shown varying levels of trypanosome infection in livestock (PNLMTN records and M. S. Kagbadouno and colleagues, 2012) [30]. Entomological and veterinary missions have identified trypanosome species in tsetse flies and mainly pigs, highlighting the need for continued monitoring potential *T. b. gambiense* animal reservoirs [31,32].

## Post-validation surveillance plan

Thanks to the efforts over the last 25 years of the PNLTHA, together with local and international partners, WHO has validated the elimination of HAT as a public health problem in Republic of Guinea in January 2025. The goal is now to achieve the elimination of transmission by 2030, according to WHO roadmap [33]. Guinea has maintained a threshold of less than 1 case per 10,000 inhabitants per district for the 2020–2024 period. The focus now shifts to long-term maintenance of elimination and prevention of resurgence by implementing two complementary strategies. Firstly, the PNLMTN-PCC deploys a targeted, reactive, personalized, and integrated medical control. Since the Ebola and Covid-19 crises, mass screenings have been replaced by proximity and participatory Door-to-Door (D2D) strategies [1]. RDT-based passive detection has been reinforced. The screening of other NTDs such as leprosy, Buruli ulcer, mycetoma, noma and yaws has been integrated during surveillance activities. Through collaboration with the National Malaria Control Program, malaria RDTs, malaria treatments, and mosquito nets are provided. Active screening is evolving towards targeted, reactive interventions based on spatial monitoring, considering patients’ activity and exposure locations [24]. These approaches minimize costs while maximizing case detection. Secondly, the PNLMTN-PCC continues to reinforce community involvement in VC. The deployment of tiny targets to control tsetse is gradually transferred to communities, supervised by the program’s entomologists. Community agents are trained in trap deployment, data collection using smartphones with the KoboCollect software and conservation of tsetse for downstream molecular detection and identification analyses. Awareness campaigns encourage individuals to use their own portable ‘companion’ tiny targets during occupational activities in transmission risk areas. Reaching the ‘zero transmission’ goal will only be possible with sustained human and financial resources, and through further integration of the PNLMTN-PCC activities into the Guinean health system, strengthening the integration will facilitate the detection of other NTDs.

The integration of research into HAT control is a strength of the Guinean model. The PNLMTN-PCC team, together with local and international partners, has conducted multiple translational and applied scientific research activities oriented towards the elimination of the disease. These research efforts are pursued on different priority topics. As recent studies have demonstrated the existence of asymptomatic carriers with a strong serological response and/or bearing extravascular parasites in the skin [34,35], the importance of human reservoirs that might escape current screening/treatment strategies is further investigated. The potential role of domestic and wild animals as reservoirs for *T. b. gambiense* is also explored [34], using a targeted metagenomic approach to identify the blood meal origin of vectors and the genetic diversity of pathogens [32]. The post-validation risk evaluation framework will include both surveillance of animal infections and skin carriage. Active prospections for blood collection will be pursued among domestic animals in ancient and active transmission foci, and metabarcoding approaches on tsetse midgut contents will be performed to identify blood feeding sources and fly infection prevalence. For monitoring skin infections in humans, rather than a tedious and invasive individual skin sampling and analysis scheme, a large-scale multiplex seroprevalence study is in preparation that will reveal the actual level of extravascular infections in populations at risk of gHAT. The PNLMTN-PCC has also been actively involved in international clinical trials on fexinidazole [23] and acoziborole [36] treatments. Acoziborole represents a marked improvement in patient management, including children, and potentially allows for the treatment of asymptomatic carriers and serological suspects, which will be crucial for interrupting transmission by 2030.

## Conclusion

Strong official framework sustained support from partners, an integrated control approach, and an active community participation constitute the strengths of the Guinean HAT elimination program. However, socio-political instability, dependence on external funding, potential hidden reservoirs may threaten its success. To address these challenges, the PNLMTN-PCC aims to secure national funding, to integrate control of NTDs, to continue research and to introduce acoziborole treatment as soon as possible. Through country ownership, the program will also strengthen its staff and systematize spatial monitoring of detected HAT patients to ensure the sustainability of control efforts.

## Methods

The very first draft of this article was generated with Le Chat (Mistral AI, France) in February 2025, by summarizing and translating each chapter of the ‘Dossier de demande de validation de l’élimination de la trypanosomiase humaine africaine comme problème de santé publique en Guinée’ written by the co-authors and validated by WHO on January 13th, 2025. This initial version was then substantially remodeled and improved over at 4 rounds of review by all the co-authors.

**Learning points**

- Combination of medical surveillance and vector control involving local communities in endemic areas was key to reach the first step of the elimination goal.
- These efforts were sustained over more than 20 years by a dedicated team of health workers as part of a national programme supported by the Ministry of Public Health and Hygiene.
- In the meantime, through multiple international collaborations, integration of research activities within public health actions allowed to progressively adapt strategies and tools to the evolving epidemiological context.

**Key papers**

- Sleeping sickness diagnosis: use of buffy coats improves the sensitivity of the mini anion exchange centrifugation test. Camara M, Camara O, Ilboudo H, Sakande H, Kaboré J, N’Dri L, Jamonneau V, Bucheton B. Trop Med Int Health. 2010 Jul;15(7):796–9. https://doi.org/10.1111/j.1365-3156.2010.02546.x. Epub 2010 May 21.PMID: 20497407
- Reducing Human-Tsetse Contact Significantly Enhances the Efficacy of Sleeping Sickness Active Screening Campaigns: A Promising Result in the Context of Elimination. Courtin F, Camara M, Rayaisse JB, Kagbadouno M, Dama E, Camara O, Traoré IS, Rouamba J, Peylhard M, Somda MB, Leno M, Lehane MJ, Torr SJ, Solano P, Jamonneau V, Bucheton B. PLoS Negl Trop Dis. 2015 Aug 12;9(8):e0003727. https://doi.org/10.1371/journal.pntd.0003727. eCollection 2015.PMID: 26267667
- Accelerating elimination of sleeping sickness from the Guinean littoral through enhanced screening in the post-Ebola context: A retrospective analysis. Camara O, Biéler S, Bucheton B, Kagbadouno M, Mathu Ndung’u J, Solano P, Camara M. PLoS Negl Trop Dis. 2021 Feb 16;15(2):e0009163. https://doi.org/10.1371/journal.pntd.0009163. eCollection 2021 Feb.PMID: 33591980
- Extravascular Dermal Trypanosomes in Suspected and Confirmed Cases of gambiense Human African Trypanosomiasis. Camara M, Soumah AM, Ilboudo H, Travaillé C, Clucas C, Cooper A, Kuispond Swar NR, Camara O, Sadissou I, Calvo Alvarez E, Crouzols A, Bart JM, Jamonneau V, Camara M, MacLeod A, Bucheton B, Rotureau B. Clin Infect Dis. 2021 Jul 1;73(1):12–20. https://doi.org/10.1093/cid/ciaa897.PMID: 32638003
- Conducting active screening for human African trypanosomiasis with rapid diagnostic tests: The Guinean experience (2016–2021). Camara O, Kaboré JW, Soumah A, Leno M, Bangoura MS, N’Diaye D, Belem AMG, Biéler S, Camara M, Bart JM, Rotureau B, Bucheton B. PLoS Negl Trop Dis. 2024 Feb 20;18(2):e0011985. https://doi.org/10.1371/journal.pntd.0011985. eCollection 2024 Feb.PMID: 38377123
